# Fast Localization and Sectioning of Mouse Locus Coeruleus

**DOI:** 10.1155/2020/4860735

**Published:** 2020-03-01

**Authors:** Song Cao, Juan Li, Jie Yuan, Dexin Zhang, Tian Yu

**Affiliations:** ^1^Department of Pain Medicine, Affiliated Hospital of Zunyi Medical University, Zunyi, 563000 Guizhou, China; ^2^Guizhou Key Laboratory of Anesthesia and Organ Protection, Zunyi Medical University, Zunyi, 563002 Guizhou, China

## Abstract

The locus nucleus (LC) is a multifunctional nucleus which is also the source of norepinephrine in the brain. To date, there is no simple and easy method to locate the small LC in brain sectioning. Here we report a fast, accurate, and easy-to-follow protocol for the localization of mice LC in frozen sectioning. After fixation and dehydration, the intact brains of adult mice were placed on a horizontal surface and vertically cut along the posterior margin of the bilateral cerebral cortex. In the coronal cutting plane, the aqueduct of midbrain can be seen easily with the naked eyes. After embedding the cerebellum part with optimal cutting temperature (OCT) compound, coronal brain slices were cut from the cutting plane, within 1 mm, the aqueduct of midbrain disappeared and the fourth ventricle appeared, then the brain slices contained LC and were collected. From the first collection, at ~200 *μ*m, the noradrenergic neurons' most enriched brain slices can be collected. The tyrosine hydroxylase immunofluorescence staining confirmed that the localization of LC with this method is accurate and the noradrenergic neuron most abundant slices can be determined with this method.

## 1. Introduction

Locus coeruleus- (LC-) related researches are important parts of neuroscience because it is not only a key hub of noradrenergic neurons, but also the only (or most important) source of norepinephrine in the brain [1]. LC projects into the brain to participate in the regulation of circadian rhythm [2], stress [3], memory and cognition [4], general anesthesia [5], and so on. LC also innervates the spinal cord to regulate physiological and pathological pain [6]. Pathologies of LC affects the cortex, hippocampus, cerebellum, and spinal cord, which can lead to depression, anxiety, Parkinson's disease, and Alzheimer's disease [7–10].

Mice are widely used experimental animals, but mouse LC is small (about 500 *μ*m in sagittal plane) [11] and lacks obvious localization landmarks, making it difficult for novices to accurately and quickly locate in brain slice sectioning. Schmidt et al. [12] provided a step-by-step protocol to localize LC in mouse brain sectioning; however, the operations are complicated. Here we report an easy-to-follow method that quickly and stably determines the cutting plane and locates the LC, as well as the noradrenergic neuron most enriched planes. We have determined the accuracy and efficiency of this method by immunofluorescence of tyrosine hydroxylase (TH) positive LC noradrenergic neurons.

## 2. Materials and Methods

### 2.1. Animals

Male and female C57BL/6N mice (*n* = 6 for each gender), weighing 20–30 g, were and purchased from Tianqin Biotechnology (Changsha, Hunan, China) and raised in the animal center of Zunyi Medical University. Mice were given 12 h light/dark cycles and free access to chows and water. This study is kept in accordance with the national medical laboratory animal management standards. The experimental procedures follow the animal experiment specifications and are approved by the Animal Ethics Committee of Zunyi Medical University ([2019]2-351).

### 2.2. Reagents and Instruments

Paraformaldehyde (Sigma, USA), sucrose (Sigma, USA), optimal cutting temperature (OCT) compound (Abcam, UK), rabbit anti-TH polyclonal antibody (Abcam, ab112), goat anti-rabbit IgG H&L (Alexa Fluor® 488, Abcam, ab150077), goat serum (Abcm, ab7481), BSA blocking buffer (Thermo Fisher, 37520, USA), Fluoroshield mounting medium (Acam, ab104135), cryostat (Leica, CM3050 S, Germany), and fluorescence microscope (Nikon, 80i, Japan).

### 2.3. Brain Collection and Preparation for Frozen Sectioning

Phenobarbital sodium (80 mg/kg) was intraperitoneally injected to anesthetize mice. The depth of anesthesia is tested by pinching the reaction of the hind paws. After the mice did not respond to the pinch, the chest was surgically opened, the heart was exposed, the right atrial appendage was cut, and a 25G needle was inserted into the left ventricle and infusion with a pump was started: firstly perfused with 10 mL of phosphate buffered saline (PBS), followed by 100 mL of 4°C precooled 4% paraformaldehyde (PFA, 20 mL/min). After decapitation, the intact brain was collected, and fixed in 4% PFA for 24 hours at 4°C, followed by dehydration in 30% sucrose solution at 4°C for 48 hours until the brain sank to the bottom of bottle.

### 2.4. Embedding Brain with OCT Compound

**Figure 1:**
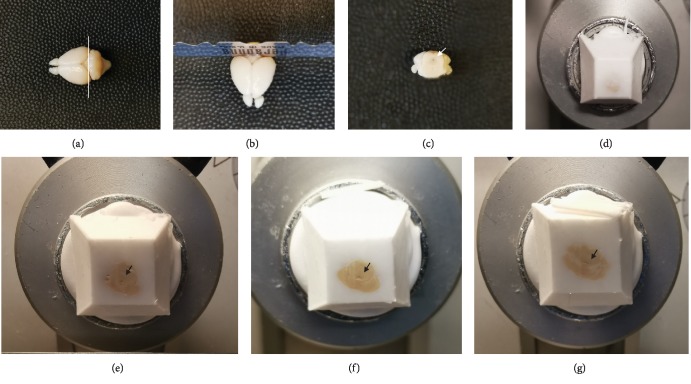
LC localization method. (a) Along the line (indicated with a white line) connecting the posterior border of the cerebral cortex on both sides, vertically cut brain with a razor blade (b) can create a coronal section (c) and separate the cerebellum part (c) from the brain part, and the midbrain aqueduct (arrows in c and e) is visible on the cutting-surface (c and e). Sectioning the cerebellum part from this cutting plane, the midbrain aqueduct (e) will disappear quickly and the fourth ventricle (4 V, arrow in f) will appear. At this point, the LC-containing brain slices can be collected when the 4 V (arrow in f) appears, and the noradrenergic neuron most enriched slices can be collected at ~200 *μ*m from the appearance of the 4V, where the 4V form a slit (g, arrow indicates the 4 V).

After fixation and dehydration, the whole brain (including the brain and cerebellum) was placed on a level surface, along the line links the posterior border of the bilateral cerebral cortex (Figure 1(a)), the brain was vertical (coronal plane in the mice brain atlas [11]) cut into the brain part, and the cerebellum part by using a razor blade (Figure 1(b)). The cerebellum part was embedded in OCT compound: inject some OCT compound to a plastic embedding mold (Thermo, 441228, USA) and put the cerebellum part of the brain in with the cutting-surface faces to the bottom of the mold and contacts the bottom, add enough OCT compound to cover all the tissue. Then, place the mold horizontally on dry ice to quickly freeze the OCT compound before remove the mold to -80°C freezer.

### 2.5. Frozen Sectioning and Brain Slice Collecting

Peel away the mold and take out the OCT compound block. The embedding block containing the cerebellum part was freeze-fixed on the chuck of the cryostat with OCT compound, and the bottom surface of the embedding block (the cutting-surface) was exposed forward (Figure 1(d)). Adjust the angle to make sure the cutting plane is parallel to the bottom surface of the embedding block, set the slice thickness to 35 *μ*m, and start the sectioning. When the midbrain aqueduct disappears, and the fourth ventricle (4V) appears, start collecting brain slices containing LC and stop the collecting when the 4V becomes a triangle shape (Bregma -6.0 mm, ~500 *μ*m from the appearance of 4V).

### 2.6. Localization Confirmation with Immunohistochemistry of LC Noradrenergic Neurons

To confirm the accuracy of LC localization, immunohistochemical detection of TH, an enzyme highly enriched in the noradrenergic neurons in LC [13] and used widely as a LC biomarker [14, 15], was conducted. The collected brain slices were (1) washed 5 mins × 3 times in PBS, (2) washed 5 mins × 3 times in PBS containing 0.3% Triton, (3) blocked for 2 hours at room temperature in TBS solution containing BSA and 2% goat serum, (4) incubated overnight at 4°C with rabbit anti-TH antibody (1:500), (5) washed 5 mins × 3 times in PBS containing 0.3% Triton, (6) incubated for 2 hours at room temperature with secondary antibody (1:500), (7) washed 5 mins × 3 times in PBS containing 0.3% Triton, and (8) stuck and sealed with mounting medium containing Fluoroshield. Then, the slices were observed and images were taken with a fluorescence microscope.

### 2.7. Statistical Analysis

The cutting distances from the cutting plane to the 4V and from the first appearance of 4V to the LC neuron most enriched plane are displayed as mean ± SD. Figures were produced with GraphPad Prism (v7.0, GraphPad Software, USA). Cutting distance comparisons between male and female group were estimated by unpaired *t* tests. *P* < 0.05 was considered as statistically significant.

## 3. Results

### 3.1. Localization of LC in Brain Tissue and Embedding Block

**Figure 2:**
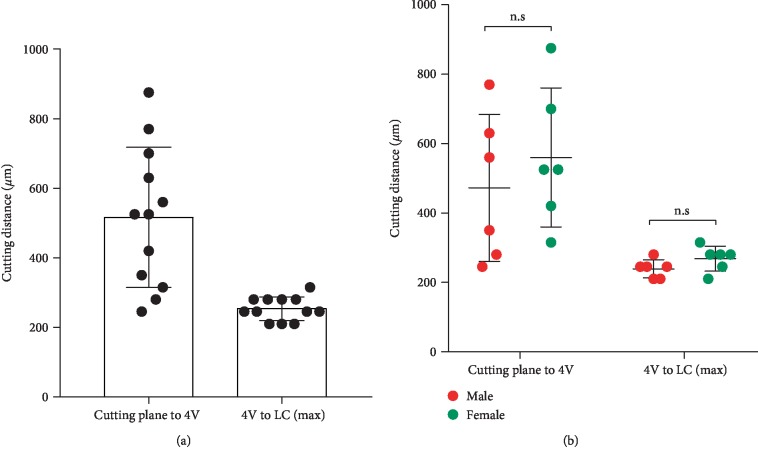
Cutting distances from the cutting plane to the 4V and from 4V to the LC neuron most enriched plane. (a) Cutting distances from the cutting plane to the 4V are within 1 mm, and from the appearance of 4V to the LC most enriched plane, the distance is ~200 *μ*m. *n* = 12. (b) Cutting distances showed no difference between male and female mice. *n* = 6, unpaired *t* tests.

Vertically cut the brain along the line cross the posterior border of the cerebral cortex on both sides (Figure 1(a)) with a razor blade (Figure 1(b)), a coronal plane [11] of the cerebellum part can be made (Figure 1(c)), and the midbrain aqueduct is visible on the cutting plane (Figures 1(c) and 1(e)). Of note, the midbrain aqueduct is close to the 4V, where the LC located nearby. Sectioning from this plane, the midbrain aqueduct (Figure 1(e)) will disappear quickly and the 4V will appear (Figure 1(f)) within 1 mm distance (Figure 2(a)). At this point, the brain slices containing LC can be collected.

### 3.2. Determine the Noradrenergic Neuron most Enriched Plane of LC

**Figure 3:**
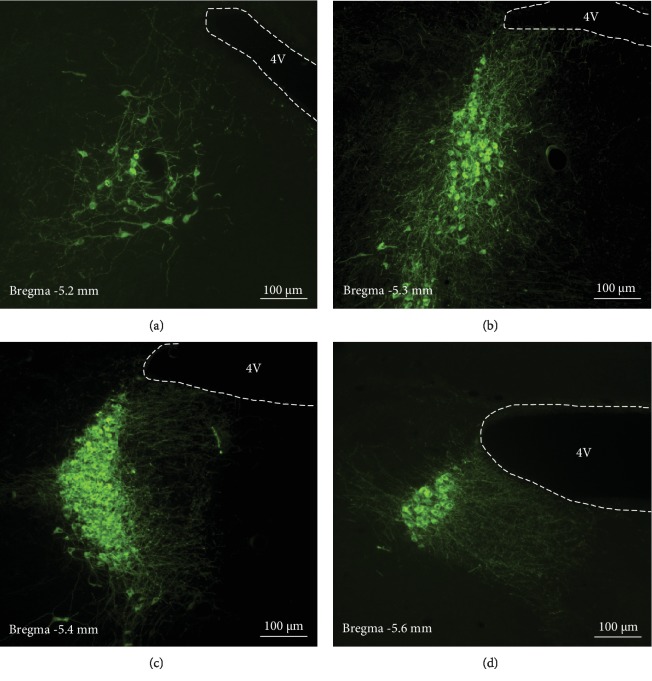
Immunofluorescence of TH to confirm accuracy of LC localization. (a–d) Brain slices were sequentially sectioned from the cutting plane (Figure 1(c)), it can be seen that the LC staining is different in different areas. The numbers and alignments of TH positive (green) noradrenergic neurons are different at different section planes. (a) Staining of rostral LC in brain slice collected when the ventricle (4V) appears (Figure 1(f)); (b) The staining of brain slice 100 *μ*m apart from the appearance of the 4V (Figure 1(f)); (c) The noradrenergic neuron most enriched brain slice collected at the level of Figure 1(g); (d) The staining of caudal LC at the planes where the 4V is vertically widened (Bregma -5.6 mm). Slice thickness = 35 *μ*m.

Brain slices were collected starting from the appearance of 4V (Figure 1(f)), and then at about 200 *μ*m (Figure 2(a)), when the 4V widened horizontally into a narrow slit (Figure 1(g)), it is the LC noradrenergic neurons' most enriched (Figure 3(c)) plane [11]. No difference was found for the cutting distances between male and female mice (Figure 2(b)).

### 3.3. Immunofluorescence of TH to Determines the Accuracy of LC Localization

Immunofluorescence for TH on different brain slices collected as described above showed accurate LC localization (Figure 3). Slices collected when the 4V first appeared show a small amount of noradrenergic neurons (Figure 3(a)), indicating the rostral part of LC; 100 *μ*m after the plane of 4V appearance, the number of TH positive neurons significantly increased, and the LC is much longer (Figure 3(b)); The brain slices collected at the noradrenergic neuron most enriched plane (Figure 1(g)) show crescent-shaped LC and the TH positive noradrenergic neurons are closely arranged in the largest number (Figure 3(c)); Then, the number of TH positive neurons gradually decreased, and the LC gradually moved to the sides of the 4V (Figure 3(d)) until it disappeared.

## 4. Discussion

This new mouse LC localization method has two major advantages: fast and easy to master. Firstly, the obvious anatomical landmarks of the caudal bilateral cerebral cortex are used to determine the coronal cutting level to assist in the separation of the brainstem and cerebellum from the cortex. Secondly, the midbrain aqueduct on the cutting surface is used as a reference point in sectioning process and help the operators to quickly reach the LC planes where the midbrain aqueduct disappears and the 4V appears. Thirdly, with this method, the noradrenergic neuron's most abundant areas of LC can be quickly located. Compared with previous reports, the localization protocol is simple and easy and can improve the accuracy and efficiency of frozen sectioning of LC in mice, and rat maybe.

The protocol reported by Schmidt et al. [12] is very detailed; however, the operation is much more complicated: (1) The cutting plane is determined with an adult mouse brain slicer matrix and at Bregma -3 mm [12]; however, there are individual differences in mouse brain volume and the degree of dehydration also affects brain volume. At the same time, the brain tissue becomes hard after dehydration with sucrose and may not fit well with the slicer matrix, so there may be large individual differences for the measure of the positioning line. (2) Besides the Bregma coordinate, there is no anatomical landmark in the process of subsequent frozen sectioning, which requires the operator to have certain sectioning experience and high concentration of attention during the frozen sectioning, and it seems difficult for novices to do this work.

In our protocol, the posterior margin of the bilateral cortex was used as a stable anatomical landmark for brain cutting before embedding, and there is no need to use slicer matrix and other positioning parameters, which minimizes the impact of individual differences and system errors on the accuracy of localization before the embedding.

After embedding with OCT compound, the midbrain aqueduct on the coronal cutting planes also served as an anatomical landmark, which will disappear within 1 mm and appear the 4V, where the LC is located. Therefore, the posterior margin of the bilateral cortex and the midbrain aqueduct are good landmarks for coronal plane cutting and frozen sectioning, respectively.

The appearance of 4V is also served as the time point for brain slice collecting. 4V also helps locate and collect the LC neuron most enriched brain slices, which are widely used to compare LC noradrenergic neuron state between groups [16, 17]. This time point is easy to master because not far from the appearance of 4V (~200 *μ*m), when the 4V turns to a line (Figure 1(g)) from a small triangle (Figure 1(f)), it is the planes with most abundant TH positive neurons in LC (Figure 1(c)).

In conclusion, this method of mouse LC localization in frozen sectioning is simple, accurate, stable and efficient, and is especially suitable for beginners of LC frozen sectioning.

## Data Availability

The data used to support the findings of this study are available from the corresponding author upon request.
